# Effect of Streptomycin Treatment on Bacterial Community Structure in the Apple Phyllosphere

**DOI:** 10.1371/journal.pone.0037131

**Published:** 2012-05-21

**Authors:** Erika Yashiro, Patricia S. McManus

**Affiliations:** Department of Plant Pathology, University of Wisconsin-Madison, Madison, Wisconsin, United States of America; Argonne National Laboratory, United States of America

## Abstract

We studied the effect of many years of streptomycin use in apple orchards on the proportion of phyllosphere bacteria resistant to streptomycin and bacterial community structure. Leaf samples were collected during early July through early September from four orchards that had been sprayed with streptomycin during spring of most years for at least 10 years and four orchards that had not been sprayed. The percentage of cultured phyllosphere bacteria resistant to streptomycin at non-sprayed orchards (mean of 65%) was greater than at sprayed orchards (mean of 50%) (*P* = 0.0271). For each orchard, a 16S rRNA gene clone library was constructed from leaf samples. Proteobacteria dominated the bacterial communities at all orchards, accounting for 71 of 104 OTUs (determined at 97% sequence similarity) and 93% of all sequences. The genera *Massilia*, *Methylobacterium*, *Pantoea*, *Pseudomonas*, and *Sphingomonas* were shared across all sites. Shannon and Simpson’s diversity indices and Pielou’s evenness index were similar among orchards regardless of streptomycin use. Analysis of Similarity (ANOSIM) indicated that long-term streptomycin treatment did not account for the observed variability in community structure among orchards (R = −0.104, *P* = 0.655). Other variables, including time of summer, temperature and time at sampling, and relative distance of the orchards from each other, also had no significant effect on bacterial community structure. We conclude that factors other than streptomycin exposure drive both the proportion of streptomycin-resistant bacteria and phylogenetic makeup of bacterial communities in the apple phyllosphere in middle to late summer.

## Introduction

The use of antibiotics in agriculture is controversial because of the possibility of selection for antibiotic resistant bacteria on farms and subsequent horizontal transfer of resistance genes to clinically important bacteria [20], [44]. Most attention has focused on the practice of adding antibiotics to animal feed, because many of the antibiotics used as growth supplements for animals are also important in the treatment of human disease [16]. However, the use of antibiotics for the control of plant diseases has drawn scrutiny ever since streptomycin was first used for this purpose in the late 1950s [7]. In the United States, the application of antibiotics to plants accounts for less than 0.5% percent of total antibiotic use, with application of streptomycin and oxytetracycline to tree fruits and nursery plants for the control of fire blight accounting for the majority of antibiotic use in plant agriculture [18]. Nevertheless, streptomycin-resistant plant pathogens, including *Erwinia amylovora*, the fire blight pathogen, have emerged. In some cases *strA* or the gene pair *strA*-*strB*, which encode aminoglycoside phosphotransferases, are carried on transposon Tn*5393* [5], [18]. Variants of Tn*5393* have been described in bacteria inhabiting diverse ecological niches, including soil, plants, animals and humans [23], [35], suggesting the possibility for further horizontal transfer of resistance genes in the environment or on food. Because of the potential environmental and human health risks associated with antibiotic use, in 2004 the European Union implemented tighter restrictions on the use of streptomycin on plants [15]. Similar reasoning led the National Organic Standards Board in the United States to recommend phasing out use of streptomycin and oxytetracycline in organic tree fruit production by 2014 (http://www.ams.usda.gov/AMSv1.0/getfile?dDocName=STELPRDC5091714).

The heightened regulations and general concerns surrounding the use of streptomycin and other antibiotics in plant agriculture have been an impetus behind studies addressing the impact of antibiotics on bacterial communities in cropping systems. Rodríguez et al. [30] found that bacterial community diversity, the proportion of bacteria resistant to streptomycin or gentamicin, and the presence of tetracycline resistance genes in bacteria on lettuce leaves did not differ among eight farms where those antibiotics were used and one non-exposed organic farm. Likewise, Rodríguez-Sánchez et al. [31] reported that use of oxytetracycline and gentamicin on coriander did not affect the abundance of bacteria resistant to those antibiotics, the presence of tetracycline- or gentamicin-resistance genes, or the presence of broad-host-range plasmids from the IncP-1 and IncQ incompatibility groups, which have been implicated in horizontal transfer of antibiotic resistance genes [26], [38]. Studies on the effects of streptomycin on bacterial communities in agricultural settings are contradictory. For example, Tolba et al. [40] compared soil from a streptomycin-treated apple orchard in Germany with non-exposed soil from the same research station and found that although the treated and non-treated soils did not differ in the proportion of bacteria resistant to streptomycin, the incidence of *strA* and *strB* was greater in treated soil. However, in a survey of soils from diverse habitats in Europe [42], the abundance of *strA*, *strB*, and genes encoding aminoglycoside nucleotidyltransferases was not greater in streptomycin-treated soil from an apple orchard in Germany compared to non-treated soil from the same location or soils from other polluted or pristine sources. Likewise, Duffy et al. [6] concluded that the use of streptomycin in orchards had no effect on indigenous bacterial communities in soil or in the phyllosphere. By contrast, the incidence of streptomycin-resistant bacteria was greater in the phyllosphere, but not the soil, of pear trees from a nursery that had received 15 sprays of streptomycin over a 2-year period compared to samples from nurseries where streptomycin had not been used [39]. These previous studies have shed light on community membership and antibiotic resistance mechanisms, but they have lacked replicated treatment and control sites, making it impossible to account for site-to-site variability in bacterial communities unrelated to antibiotic exposure.

In the current study we test the hypothesis that the use of streptomycin in commercial apple orchards alters bacterial community structure on apple leaves. We compared phyllosphere bacterial communities in four commercial apple orchards where streptomycin had been used for several years with communities from four orchards where streptomycin had not been used. Our goal was to assess the long-term effects of streptomycin use, rather than the transient disruption of communities that might be expected in the spring immediately after spraying. To this end, we sampled leaves in middle to late summer, constructed 16S rRNA clone libraries from phyllosphere bacteria, identified operational taxonomic units (OTUs) from the cloned sequences, and then used diversity indices to describe communities. Because certain bacterial taxa have previously been identified as likely reservoirs of antibiotic-resistance genes in the phyllosphere [17], [19], [33], [34], it was relevant to identify bacterial taxa in orchards, an objective served better by obtaining 16S rRNA gene sequences than fingerprinting methods. We used UniFrac and analysis of similarity (ANOSIM) to further test the effects of streptomycin treatment and other variables on the phylogenetic makeup of bacterial communities.

## Materials and Methods

### Ethics Statement

No specific permits were required for the described field studies. At each study site, the landowner granted us permission to collect apple leaves. The studies did not involve endangered or protected species.

### Sample Collection

Apple leaves were collected in southern Wisconsin, USA, at eight commercial orchards during early July through early September, 2009 (Table 1). At all sites, fungicides, insecticides, and fertilizers were applied as needed to maintain tree health. At four orchards (Ep, SR, BFF, and LP) the sampled trees were sprayed with streptomycin sulfate at a concentration of 50 to 100 ppm up to three times annually during the bloom period (early to mid May) for at least the past 10 years. However, orchard BFF was not sprayed in 2008, and orchards BFF and LP were not sprayed in 2009, the year of sampling, because weather conditions were not conducive to fire blight. At three orchards (DC, EL, and GPS) streptomycin had never been used, while at one orchard (BW) streptomycin was sprayed once prior to 1995. Despite this one spray, orchard BW was classified as “not sprayed” in this study. At the time of streptomycin application, leaves were expanding. None of the orchards was treated with oxtetracycline, another antibiotic registered for use on apple trees.

**Table 1:** Sampling sites and conditions.

			GPS coordinates				
Orchard	Streptomycin treatment^a^	Date of last streptomycin spray	Latitude	Longitude	Sampling date	Time of day^b^	Air temp (°C)
Ep	sprayed	14 May–09	42.974	−89.475	6 Jul–09	1100	27
SR	sprayed	15 May–09	43.317	−90.833	29 Jul–09	0955	29
BFF	sprayed	14 May–07	43.242	−88.026	14 Aug–09	1024	28
LP	sprayed	May–08^c^	43.348	−89.287	1 Sep–09	1100	20
DC	not sprayed	–	43.025	−89.221	8 Jul–09	0900	16
EL	not sprayed	–	42.745	−88.231	21 Jul–09	1049	25
GPS	not sprayed	–	42.997	−89.209	5 Aug–09	0950	24
BW^d^	not sprayed	Before 1995	42.644	−88.162	12 Aug–09	1035	NA^c^

a “Sprayed” orchards were sprayed with streptomycin sulfate at a concentration of 50 to 100 ppm up to three times annually during the bloom period (early to mid May) for at least the past 10 years. “Not sprayed” orchards were not sprayed with streptomycin.

b Central Daylight Time.

c Day of last spray at orchard LP is unknown.

d Temperature data not available at orchard BW.

At each orchard, 50 fully-expanded, healthy-appearing leaves from each of five or six arbitrarily selected trees (i.e., a total of 250 or 300 leaves per orchard) of the cultivar ‘Gala’ were collected at least 2 days after the last rainfall to minimize the effect of rain on bacterial community structure [10]. Leaves were collected from throughout the tree canopy by their petioles with gloved hands, packed individually in sterile plastic bags, stored on ice during transport to the laboratory, and then stored for up to 2 days at 4°C until processed. In the laboratory, using sterile tweezers and scissors, petioles were removed and discarded. Leaf blades were weighed individually and then sonicated in 10- to 13-leaf batches in 400 ml of sterile deionized water containing approximately two to three drops Tween 20 per liter in a tabletop ultrasonic cleaning bath (Branson model 3510, Branson Ultrasonic Corp., Danbury, CN, USA) for 5 min. The extracts from all the leaves from a single tree were bulked.

### Bacterial Enumeration

An aliquot of the combined leaf extracts from each tree was serially diluted and then plated onto 0.1× tryptic soy agar (TSA) amended with cycloheximide (100 µg ml^−1^) to inhibit fungal growth, as well as 0.1× TSA amended with cycloheximide (100 µg ml^−1^) and streptomycin sulfate (50 µg ml^−1^) to select for streptomycin resistant bacteria. After incubation for 7 days at room temperature and with ambient light, bacterial colonies were counted and the proportion resistant to streptomycin was calculated. A two-tailed Student’s t-test was calculated on the mean percent streptomycin resistance between sprayed and non-sprayed orchards to determine the long-term effect of streptomycin use on bacterial populations. To examine the shorter term effect of streptomycin on the leaf bacterial community, colony counts from the 10 individual trees from the orchards that were sprayed in the sampling year (i.e., Ep and SR) were combined into a single dataset, while colony counts from the 10 trees from orchards that were not sprayed in the sampling year but had a history of streptomycin exposure (i.e., BFF and LP) were combined. A two-tailed Student’s t-test was then calculated, with N = 10 for sprayed in sampling year and N = 10 for not sprayed in sampling year.

### DNA Extraction and 16S rRNA Gene Clone Library Construction

Bacterial cells in leaf extracts from each tree were pelleted by centrifugation at 12,857×g at 4°C for 15 min. Pellets were then resuspended in residual supernatant and centrifuged at 16,870×g for 5 min in a microcentrifuge to further concentrate the bacteria. Genomic DNA was extracted from each individual cell pellet using the FastDNA spin kit (MP Biomedicals, Solon, OH, USA). PCR amplification of 16S rRNA genes was performed in triplicate using primers 27f and 1492r as described in Bräuer et al. [4] on two (orchard BFF) or four (all other orchards) extracts for a total of six or 12 independent reactions per orchard. Amplification products were combined for each orchard, purified using the Wizard SV Gel and PCR Clean-up System (Promega, Madison, WI, USA), and then cloned into the pGEM-T Easy vector according to the manufacturer’s instructions (Promega). *Escherichia coli* DH5α cells were transformed with the ligation products, and a clone library was constructed for each orchard. The 16S rRNA gene insert of each clone was amplified using the M13f(-20) and M13r(-27) primers according to Bräuer et al. [4] and sequenced from the 5′ end using the 27f primer on an Applied Biosystems 3730xl automated DNA sequencing instruments (Applied Biosystems, Foster City, CA, USA) at the University of Wisconsin-Madison Biotechnology Center.

### Taxonomic and Statistical Analyses of Bacterial Communities

The sequences were screened against chloroplast and other non-ribosomal DNA using BLASTn and the Ribosomal Database Project Classifier [1], [45]. Potential chimeras were identified using Mallard and omitted from further study after manual verification [2]. Short sequences that did not cover the reference *E. coli* base positions 103 to 920 were also omitted from further study. The remaining sequences were aligned using the SILVA aligner (25) and imported into ARB [14]. Using a filter to exclude the nonoverlapping ends beyond positions 103 to 920, a neighbor-joining matrix was generated in ARB and exported. The fasta file from these same sequences was also exported and used for taxonomic classification of the bacterial communities using the RDP Classifier.

Mothur [32] was used to assign operational taxonomic units (OTUs) at the 97% sequence similarity level and to calculate Shannon and Simpson’s diversity indices, Pielou’s index of evenness, and rarefaction curves. For beta diversity analyses, PhyML was used to generate a maximum likelihood tree of all the sequences [8]. The settings used were: the general time reversible (GTR) correction model; optimized equilibrium frequencies; estimated proportion of invariable sites, four substitution rate categories; estimated gamma shape parameter; and SPR tree topology search operations. The validity of using the GTR correction model was also confirmed by applying jModelTest2 on smaller subsets of the 16S rRNA gene dataset [24]. Due to the computer intensive nature of the software, the full dataset was not processed. The tree generated by PhyML was then imported into mothur, and the weighted UniFrac metric that accounts for phylogenetic relationships among taxa [13] was calculated and visualized in a Principle Coordinate Analysis (PCoA) plot. Hypothesis testing to determine which variables influenced bacterial community structure at the different orchards was performed on weighted UniFrac using the analysis of similarity (ANOSIM) with 10,000 iterations. A Mantel test with the Pearson correlation coefficient and 1000 iterations was used to determine the effect of relative distances between orchard sites on the bacterial communities at each sampling site. The hypothesis tests were repeated using Bray-Curtis distance (non-phylogenetic) defining OTUs at the 97%, 99%, and 100% (i.e., unique sequences) similarity thresholds. The Bonferroni correction was applied to correct for multiple comparisons.

### Nucleotide Sequence Accession Numbers

The 16S rRNA gene sequences obtained in this study were deposited into the NCBI GenBank database under accession numbers JQ046416 to JQ048217.

## Results

### Proportion of Bacterial Communities Resistant to Streptomycin

At sprayed orchards 43% to 59% (mean of 50%) of the total culturable bacteria were resistant to streptomycin, whereas at non-sprayed orchards 57% to 72% (mean of 65%) of the total culturable bacteria were resistant to streptomycin (Table 2). A two-tailed Student’s t-test indicated that the proportion of streptomycin resistant bacteria differed between the sprayed and non-sprayed orchards (*P* = 0.0271). Among sprayed orchards, the observation of higher CFU counts at orchards sprayed in 2009, the year of sampling (Ep and SR), versus those not sprayed in 2009 (BFF and LP), prompted an analysis of shorter-term effects of streptomycin on total CFU. The mean log_10_ CFU/g leaf tissue was 5.58 and 6.29 for sprayed and non-sprayed orchards, respectively. A two-tailed Student’s t-test indicated that the difference in total CFU was highly significant (*P*<0.001).

**Table 2:** Bacterial CFU recovered from the apple phyllosphere and percentage that were resistant to streptomycin (strR).

Orchard	Streptomycin treatment	Log_10_ CFU/g leaf (SEM)^a^	Log_10_ strR CFU/g leaf (SEM)^b^	% strR (SEM)^c^
Ep	sprayed	5.59 (0.05)	5.36 (0.04)	59 (3.4)
SR	sprayed	5.58 (0.07)	5.21 (0.08)	43 (2.4)
BFF	sprayed	6.43 (0.13)	6.13 (0.15)	50 (2.7)
LP	sprayed	6.14 (0.13)	5.82(0.14)	49 (4.7)
Mean				50 (3.3)
DC	not sprayed	5.65 (0.03)	5.49 (0.08)	72 (10.7)
EL	not sprayed	6.58 (0.10)	6.42 (0.09)	69 (5.3)
GPS	not sprayed	6.43 (0.14)	6.22 (0.14)	61 (3.8)
BW	not sprayed	5.63 (0.09)	5.38 (0.11)	57 (3.3)
Mean				65 (3.6)
*P* d				0.0271

a CFU on TSA amended with cycloheximide. Values in rows with an orchard designation are the mean and SEM of five or six trees per orchard.

b CFU on TSA amended with cycloheximide and streptomycin. Values in rows with an orchard designation are the mean and SEM of five or six trees per orchard.

c The mean % strR and SEM for the individual orchards was calculated across trees at each orchard (N = 5 or 6). The mean and SEM for the mean % strR was calculated separately for sprayed (N = 4) and not sprayed orchards (N = 4).

d *P*-values for the two-tailed t-test comparing sprayed (N = 4) and not sprayed (N = 4) orchards.

### Phylogenetic Characterization of Phyllosphere Bacterial Communities

**Figure 1:**
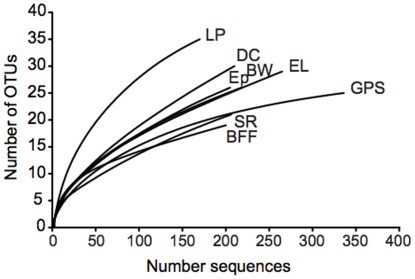
Rarefaction curves of the sequenced clones for each orchard. The sequences were binned into OTUs at the 97% similarity threshold.

A total of 3199 16S rRNA gene clone sequences were obtained for the eight-orchard study. After excluding non-ribosomal DNA (notably chloroplast DNA), low quality or short sequences, and chimeras, 1802 sequences remained for analyses, with a range of 169 to 335 sequences per orchard (Table 3; Fig. 1). Across all orchards, 104 OTUs were identified, with a range of 19 to 35 OTUs per orchard (Table 3; Fig. 1). The phylum Proteobacteria comprised 93% of the total bacterial community, followed by Bacteroidetes, Actinobacteria, Firmicutes, Acidobacteria, Tenericutes, and Chlorflexi (Table 4). Sequences for five genera, *Massilia*, *Methylobacterium*, *Pantoea*, *Pseudomonas*, and *Sphingomonas*, were shared across all sites (Table 5). At individual orchards, the predominant genus was *Sphingomonas* (orchards Ep, BFF, LP, EL, and GPS), *Pseudomonas* (orchards SR and DC), or *Massilia* (BW) (Table 5). Because the two most abundant genera, *Sphingomonas* and *Pseudomonas*, frequently possess either intrinsic or acquired streptomycin resistance [11], [19], [34], [39], [41], we tested whether the abundance of these genera varied between sprayed and not sprayed orchards. The percentage of sequences representing *Sphingomonas* in sprayed and non-sprayed orchards was 31% (range 16 to 39%) and 37% (range 6 to 64%), respectively (Table S1). The percentage of sequences representing *Pseudomonas* in sprayed and non-sprayed orchards was 23% (range 10 to 37%) and 17% (range 5 to 38%), respectively (Table S1). Two-tailed Student t-tests revealed no difference in the proportions of *Sphingomonas* (*P* = 0.7470) or *Pseudomonas* (*P* = 0.5165) at orchards sprayed with streptomycin compared to non-sprayed orchards. Table S1 shows the phylogenetic characterization and abundance of all bacterial 16S rRNA gene sequences in this study.

**Table 3:** Diversity and evenness estimates for 16S rRNA gene libraries derived from phyllosphere bacteria of apple orchards differing in exposure to streptomycin.

Orchard	Streptomycin treatment	No. sequences	No. OTUs^a^	Shannon^b^	Simpson’s 1-D^b^	Pielou’s evenness
Ep	sprayed	204	26	2.12 (0.17)	0.83 (0.02)	0.40
SR	sprayed	205	21	1.84 (0.16)	0.78 (0.03)	0.34
BFF	sprayed	199	19	2.10 (0.15)	0.83 (0.03)	0.40
LP	sprayed	169	35	2.92 (0.17)	0.92 (0.02)	0.57
Mean for sprayed		194	25	2.25	0.84	0.43
DC	not sprayed	209	30	2.07 (0.19)	0.79 (0.04)	0.39
EL	not sprayed	264	29	2.02 (0.17)	0.76 (0.04)	0.36
GPS	not sprayed	335	25	1.66 (0.17)	0.62 (0.06)	0.28
BW	not sprayed	217	26	2.06 (0.18)	0.78 (0.04)	0.38
Mean for not sprayed		257	28	1.95	0.74	0.35
*P* c		0.109	0.582	0.293	0.083	0.225

a Number of unique OTUs determined at the 97% sequence similarity threshold.

b 95% confidence intervals for the Shannon and Simpson’s indices are indicated between parentheses. Simpson’s index (D) is presented as 1-D; higher values indicate greater diversity.

c *P*-values for the two-tailed t-test comparing sprayed (N = 4) and not sprayed (N = 4) orchards.

**Table 4:** Taxonomy and abundance of bacterial 16S rRNA gene sequences in libraries constructed from apple leaves from orchards differing in streptomycin treatment.

			Orchard streptomycin treatment and no. sequences							
			Sprayed				Not sprayed			
Phylogenetic group	No. OTUs^a^	No. sequences(% of total)	Ep	SR	BFF	LP	DC	EL	GPS	BW
Protobacteria	71	1677 (93)	193	203	188	153	193	246	319	182
Alpha	28	801	71	37	127	94	21	175	228	48
Beta	14	346	34	58	10	24	28	55	37	100
Delta	6	10	3	1	2	1	3	0	0	0
Gamma	23	520	85	107	49	34	141	16	54	34
Bacteroidetes	11	57 (3)	3	0	6	12	9	15	8	4
Actinomycetes	12	31 (2)	5	2	5	4	1	3	8	3
Firmicutes	4	28 (2)	1	0	0	0	2	0	0	25
Acidobacteria	3	4 (<1)	1	0	0	0	1	0	0	2
Tenericutes	1	3 (<1)	0	0	0	0	3	0	0	0
Chlorflexi	2	2 (<1)	1	0	0	0	0	0	0	1
Total	104	1802 (100)	204	205	199	169	209	264	335	217

a Number of unique OTUs determined at the 97% sequence similarity threshold.

**Table 5:** Genera shared across all orchards.

			Orchard streptomycin treatment and no. sequences							
			Sprayed				Not sprayed			
Phylogenetic group	No. OTUs^a^	No. sequences(% of total)^b^	Ep	SR	BFF	LP	DC	EL	GPS	BW
*Sphingomonas*	13	666 (37)	64	32	77	65	13	158	216	41
*Pseudomonas*	9	345 (19)	45	75	48	16	80	14	43	24
*Massilia*	2	312 (17)	28	53	9	21	26	52	34	89
*Pantoea*	2	146 (8)	39	32	1	5	57	1	2	9
*Methylobacterium*	3	83 (5)	7	4	24	22	3	8	9	6

a Number of unique OTUs determined at the 97% sequence similarity threshold.

b Total number of sequences, including sequences for OTUs that were not shared across all orchards, was 1802.

Rarefaction curves indicated that more OTUs would have been obtained with additional sequencing (Fig. 1). Shannon and Simpson’s indices indicated that bacterial diversity was generally similar across sites, although the community from streptomycin-sprayed orchard LP was more diverse than other communities (Table 3). Pielou’s evenness index indicated that the bacterial communities were comprised of a few highly abundant taxa and many relatively rare taxa (Table 3). Two-tailed Student’s t-tests indicated no differences in Shannon or Pielou’s indices for communities from sprayed orchards compared to non-sprayed orchards (*P* = 0.293 for Shannon; *P* = 0.225 for Pielou’s), whereas the Simpson’s index indicated somewhat greater diversity (*P* = 0.083) for communities from sprayed orchards compared to non-sprayed orchards.

### Beta Diversity

**Figure 2:**
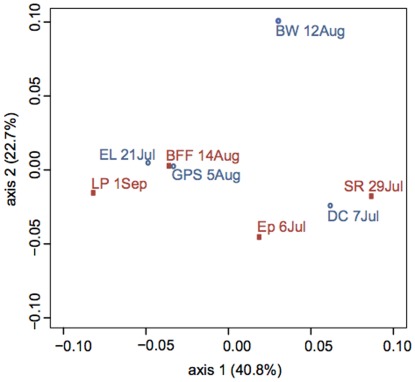
PCoA plot of weighted Unifrac generated from a maximum likelihood tree. Red squares indicate orchards that were sprayed with streptomycin; blue circles indicate orchards that were not sprayed.

The weighted Unifrac metric was used to calculate the pairwise distances between the phyllosphere bacterial communities of the eight orchards. The first two axes of the principal coordinates analysis (PCoA) represented 40.8% and 22.7%, respectively, of the total variation (Fig. 2). ANOSIM tests showed that none of the variables we tested accounted for the variability in community structure among orchards. There was no significant effect of streptomycin treatment (R = −0.104, *P* = 0.655), nor an effect of not spraying streptomycin during the sampling year regardless of long-term treatment (R = −0.177, *P* = 0.246). Although sampling of the eight orchards extended from early July to early September, the effect of sampling time did not explain the variability in community structure (ANOSIM groupings by month R = 0.198, *P* = 0.204; groupings by 2-week period R = 0.043, *P* = 0.657). The time of day of sampling, which ranged from 0900 to 1100 hr, did not account for community structure (R = 0.041, *P* = 0.398), nor did the ambient temperature at the time of sampling, which ranged from 16°C to 29°C (R = 0.094, *P* = 0.606). For total log_10_ CFU, R = 0.625 and *P* = 0.030 (Bonferroni-corrected alpha value = 0.0056). For log_10_ CFU on streptomycin-amended TSA, R = 0.056 and *P* = 0.594. Geographic proximity among the orchards also did not influence the community structure (Mantel r = 0.000, *P* = 0.639). The same conclusions (i.e., no significance for any of the variables) were obtained using Bray-Curtis distance, a non-phylogenetic metric of community similarity (Table S2).

Recalculating the weighted Unifrac after removing *Sphingomonas* from the dataset did not reveal any effects of streptomycin. The bacterial communities at orchards Ep, DC, and SR clustered together, while orchards EL and GPS became more distant from LP and BFF on a PCoA plot (Fig. S1). Closer examination revealed that *Sphingomonas* comprised 60% and 64% of the communities at EL and GPS, respectively, compared to 6% to 39% at the other sites (Table S1). Recalculating the weighted Unifrac values after removing both *Sphingomonas* and *Pseudomonas* from the dataset also did not reveal any effect of streptomycin on the remaining bacterial community (Fig. S2; Table S3). Orchard BW remained an outlier on the PCoA plot, indicating that *Sphingomonas* was not responsible for the divergence in community structure for that site. Unique to orchard BW were 25 sequences belonging to a single OTU in the phylum Firmicutes, genus *Lactobacillus* (Table 3; Table S1). Also, sequences representing the genus *Massilia* were more abundant for orchard BW than other orchards (Table 5). If orchard BW was considered an outlier, recalculating ANOSIM tests resulted in R = 0.3904 and *P* = 0.046 (Bonferroni-corrected alpha value = 0.005) for the effect of spraying during the spring of the sampling year on bacterial communities in mid to late summer.

## Discussion

The results indicate that the routine springtime use of streptomycin in apple orchards for the control of fire blight disease does not have long-term effects on the diversity or phylogenetic composition of bacterial communities in the phyllosphere in middle to late summer. However, the proportion of cultured bacteria resistant to streptomycin was lower in orchards with a history of streptomycin exposure than in non-exposed orchards. We hypothesized that this unexpected result might be explained by a chance higher abundance of *Sphingomonas* and *Pseudomonas* in non-exposed orchards, since these genera are known to have a high level of stable, intrinsic or acquired resistance to streptomycin regardless of exposure [11], [19], [34], [39], [41]. In fact, we previously observed that the order Sphingomonadales accounted for 100% of cultured streptomycin-resistant bacteria at a research orchard where streptomycin had never been used [46]. Although we did not identify cultured bacteria in the current study, we estimated the abundance of *Sphingomonas* and *Pseudomonas* based on their abundance in clone libraries. The validity of this approach is supported by the finding that the relative abundance of each of these genera was similar among cultured isolates and 16S rRNA gene clones derived from the same field samples [47]. In the present study, the abundance of *Sphingomonas* and *Pseudomonas* sequences in clone libraries was similar between the sprayed and non-sprayed orchards, making it unlikely that those genera accounted for the difference in the percentage of streptomycin-resistant colonies. We previously found a greater abundance of Actinomycetales, specifically the genera *Curtobacterium* and *Frigoribacterium*, in the apple phyllosphere by culturing on TSA than by cloning 16S rRNA genes [47]. These genera, or other members of the Actinomycetales that are known producers of streptomycin [12], [43] and therefore would also be resistant to streptomycin, might have been more abundant among the cultured bacteria than is suggested by clone libraries. However, there is no obvious reason to expect that they would be more abundant in non-sprayed than sprayed orchards. Thus, while we cannot explain the higher level of resistance in non-sprayed orchards, it is clear that springtime use of streptomycin over many years does not lead to elevated levels of streptomycin resistance in the apple phyllosphere later in the summer.

Despite a lack of long-term effects of streptomycin on bacterial phyllosphere communities, where the antibiotic was applied during spring of the year of sampling, the total bacterial population, but not the proportion of bacteria resistant to streptomycin, was reduced in mid-summer. When site BW, an outlier on PCoA plots due to observable differences in its bacterial community composition, was treated as an outlier in ANOSIM, we found weak evidence that exposure to streptomycin during the year of sampling affected the phylogenetic composition of communities.

The enterobacterium *Pantoea agglomerans* (formerly *Erwinia herbicola*) has been cited as a likely reservoir of mobile streptomycin resistance genes in apple orchards [18]. From a public health perspective, the presence of enterobacteria in orchards and on other food crops has received attention [3], [21], [30] because this group includes several food-borne pathogens as well as commensal bacteria that might serve as reservoirs for resistance genes in the human gut. In the current study, *Pantoea* was abundant in one non-sprayed and two sprayed orchards, and therefore was not obviously affected by long-term streptomycin use. We had expected a greater abundance of *Pantoea* and the related enterobacterial genera *Erwinia* and *Enterobacter* across all orchards, since these genera are commonly isolated from apple tissues during spring [17], [33]. On leaves of cottonwood, the abundance of members of the Enterobacteriacae was highly variable throughout the summer, with *Pantoea* dominating in early summer and diminishing by fall [29]. Thus, differences in the succession of bacterial communities, and the impacts of immigration, emigration, growth and death might explain the imbalance in the distribution of *Pantoea* among orchards.

Previous studies concerning the use of antibiotics in crop production report the proportion of antibiotic resistant bacteria in leaf, flower, or soil samples relative to antibiotic exposure, and some provide information on the presence of resistance genes and/or potential resistance plasmids [6], [17], [19], [30], [31], [33], [34], [36], [37], [39], [40], [42]. Our study is unique, however, in that comparable treatment and control sites were replicated, making it possible to perform statistical comparisons between sprayed and non-sprayed sites and thereby determine the effects of many years of streptomycin exposure under conditions typical in commercial apple production. Moreover, past studies aimed at identifying potential reservoirs for antibiotic resistance genes in the phyllosphere have relied on culturing [5], [17], [19], [33], [34], [39]. It is well established that culturing generally reveals only a small fraction of bacterial diversity [9], [22], [27], and recently we demonstrated greater richness of bacteria in the apple phyllosphere determined by 16S rRNA clone libraries than by culturing [47]. The current study provides the first culture-independent assessment of bacterial communities in apple orchards varying in exposure to streptomycin.

Our analyses were based on a modest number of sequences (1802 across eight orchards) relative to the thousands of sequences per sample that are routinely obtained through modern high-throughput technology. Indeed, rarefaction curves suggested that further sequencing would have revealed greater richness in our samples. While we cannot dismiss the potential ecological importance of rare taxa that might have been detected through deeper sequencing, the dominance in our dataset of a few taxa common to all sites makes it unlikely that deeper sequencing would have revealed significant differences in bacterial communities at sprayed and non-sprayed orchards, at least when analyzed by methods that take into account sequence abundance (e.g., weighted UniFrac).

We conclude that long-term streptomycin use does not increase the frequency of resistant bacteria or disrupt bacterial communities on apple leaves. Our findings contribute to a growing body of literature that indicates using streptomycin to control fire blight has low environmental impact [6], [28]. However, our conclusion does not absolve streptomycin of all risk associated with its use. For example, it is possible that streptomycin could select for novel resistance genes in apple orchards, even if the overall frequency of resistant bacteria is not increased. A greater diversity of mobile resistance genes in apple orchards could lead to horizontal transfer of resistance among a greater range of bacteria, which in turn could be consumed on fresh produce. However, for those concerned with regulating the use of streptomycin on crops, it will be critical to compare data from replicated treated and control sites, as was done in the current study, since resistance genes and mobile genetic elements are present even in environments with no known anthropogenic selection pressure [19], [40], [42].

## Supporting Information

Figure S1 **Weighted Unifrac PCoA plot of the orchard bacterial communities, excluding** ***Sphingomonas*** **species.** The streptomycin treated sites were Ep, SR, BFF, and LP. The nontreated sites were DC, EL, GPS, and BW.
(DOC)

Figure S2 **Weighted Unifrac PCoA plot of the orchard bacterial communities, excluding** ***Sphingomonas*** **and** ***Pseudomonas*** **species.** The streptomycin treated sites were Ep, SR, BFF, and LP. The nontreated sites were DC, EL, GPS, and BW.
(DOC)

Table S1 **Phylogenetic characterization and abundance of bacterial 16S rRNA gene sequences in libraries constructed from apple leaves differing in streptomycin treatment.**
(DOC)

Table S2 **ANOSIM and Mantel values from Bray-Curtis matrix of the bacterial communities at the 100%, 99%, and 97% similarity threshold.** The Bonferroni corrected alpha is 0.0056.
(DOC)

Table S3 **ANOSIM and Mantel values from weighted Unifrac matrix for the bacterial communities excluding** ***Sphingomonas*** **and** ***Pseudomonas*** **species.** The Bonferroni corrected alpha is 0.0056.
(DOC)
