# Supplementary material for: Effect of Streptomycin Treatment on Bacterial Community Structure in the Apple Phyllosphere
Source: PLoS One. 2012 May 21;7(5):e37131. doi: 10.1371/journal.pone.0037131 (PMC3357425; doi:10.1371/journal.pone.0037131)
Supplement: Figure S2 — Weighted Unifrac PCoA plot of the orchard bacterial communities, excluding Sphingomonas and Pseudomonas species. The streptomycin treated sites were Ep, SR, BFF, and LP. The nontreated sites were DC, EL, GPS, and BW. (DOC) [file pone.0037131.s002.doc]

**Supporting Figure 2.** Weighted Unifrac PCoA plot of the orchard bacterial communities, excluding *Sphingomonas* and *Pseudomonas* species. The streptomycin treated sites were Ep, SR, BFF, and LP. The nontreated sites were DC, EL, GPS, and BW.
