# Supplementary material for: Effect of Streptomycin Treatment on Bacterial Community Structure in the Apple Phyllosphere
Source: PLoS One. 2012 May 21;7(5):e37131. doi: 10.1371/journal.pone.0037131 (PMC3357425; doi:10.1371/journal.pone.0037131)
Supplement: Table S1 — Phylogenetic characterization and abundance of bacterial 16S rRNA gene sequences in libraries constructed from apple leaves differing in streptomycin treatment. (DOC) [file pone.0037131.s003.doc]

**Supporting Table 1.** Phylogenetic characterization and abundance of bacterial 16S rRNA gene sequences in libraries constructed from apple leaves differing in streptomycin treatment.

| Phylogenetic group | |  |  | Orchard streptomycin treatment and no. sequences | | | | | | | | |
| --- | --- | --- | --- | --- | --- | --- | --- | --- | --- | --- | --- | --- |
|  |  |  |  | Sprayed | | | |  | Not sprayed | | | |
|  |  | No. OTUs^a^ | No. sequences | Ep | SR | BFF | LP |  | DC | EL | GPS | BW |
| Protobacteria |  |  |  |  |  |  |  |  |  |  |  |  |
| Alpha | *Acidosoma* | 1 | 3 | 0 | 0 | 0 | 1 |  | 1 | 1 | 0 | 0 |
|  | *Afipia* | 1 | 1 | 0 | 0 | 0 | 0 |  | 0 | 0 | 1 | 0 |
|  | *Anaplasma* | 1 | 1 | 0 | 0 | 1 | 0 |  | 0 | 0 | 0 | 0 |
|  | *Blastomonas* | 1 | 1 | 0 | 0 | 0 | 0 |  | 0 | 0 | 1 | 0 |
|  | *Geminicoccus* | 1 | 1 | 0 | 0 | 0 | 0 |  | 0 | 0 | 0 | 1 |
|  | *Methylobacterium* | 3 | 83 | 7 | 4 | 24 | 22 |  | 3 | 8 | 9 | 6 |
|  | *Methylovirgula* | 1 | 14 | 0 | 0 | 2 | 4 |  | 2 | 6 | 0 | 0 |
|  | *Novosphingobium* | 1 | 3 | 0 | 1 | 0 | 1 |  | 1 | 0 | 0 | 0 |
|  | *Orientia* | 1 | 2 | 0 | 0 | 0 | 0 |  | 0 | 0 | 2 | 0 |
|  | *Pedomicrobium* | 1 | 1 | 0 | 0 | 0 | 0 |  | 0 | 1 | 0 | 0 |
|  | *Porphyrobacter* | 1 | 1 | 0 | 0 | 0 | 0 |  | 1 | 0 | 0 | 0 |
|  | *Rhodoblastus* | 1 | 1 | 0 | 0 | 0 | 0 |  | 1 | 0 | 0 | 0 |
|  | *Rickettsia* | 1 | 23 | 0 | 0 | 23 | 0 |  | 0 | 0 | 0 | 0 |
|  | *Sphingomonas* | 13 | 666 | 64 | 32 | 77 | 65 |  | 13 | 158 | 216 | 41 |
| Beta | *Burkholderia* | 2 | 3 | 0 | 0 | 1 | 1 |  | 0 | 0 | 0 | 1 |
|  | *Delftia* | 1 | 1 | 1 | 0 | 0 | 0 |  | 0 | 0 | 0 | 0 |
|  | *Massilia* | 2 | 312 | 28 | 53 | 9 | 21 |  | 26 | 52 | 34 | 89 |
|  | *Methylibium* | 1 | 4 | 2 | 1 | 0 | 0 |  | 0 | 0 | 0 | 1 |
|  | *Naxibacter* | 4 | 9 | 0 | 4 | 0 | 2 |  | 0 | 2 | 0 | 1 |
|  | *Neisseria* | 1 | 5 | 0 | 0 | 0 | 0 |  | 0 | 0 | 0 | 5 |
|  | *Oxalicibacterium* | 1 | 1 | 0 | 0 | 0 | 0 |  | 0 | 0 | 0 | 1 |
|  | *Simplicispira* | 1 | 6 | 2 | 0 | 0 | 0 |  | 0 | 1 | 1 | 2 |
|  | *Variovorax* | 1 | 5 | 1 | 0 | 0 | 0 |  | 2 | 0 | 2 | 0 |
| Delta | *Anaeromyxobacter* | 2 | 3 | 0 | 0 | 1 | 0 |  | 2 | 0 | 0 | 0 |
|  | *Cystobacter* | 1 | 1 | 0 | 1 | 0 | 0 |  | 0 | 0 | 0 | 0 |
|  | *Hyalangium* | 2 | 5 | 3 | 0 | 0 | 1 |  | 1 | 0 | 0 | 0 |
|  | *Melittangium* | 1 | 1 | 0 | 0 | 1 | 0 |  | 0 | 0 | 0 | 0 |
| Gamma | *Azomonas* | 4 | 6 | 1 | 0 | 0 | 3 |  | 1 | 0 | 1 | 0 |
|  | *Azorhizophilus* | 1 | 1 | 0 | 0 | 0 | 0 |  | 0 | 1 | 0 | 0 |
|  | *Brenneria* | 2 | 5 | 0 | 0 | 0 | 0 |  | 1 | 0 | 4 | 0 |
|  | *Cedecea* | 1 | 1 | 0 | 0 | 0 | 1 |  | 0 | 0 | 0 | 0 |
|  | *Obesumbacterium* | 1 | 4 | 0 | 0 | 0 | 0 |  | 0 | 0 | 4 | 0 |
|  | *Pantoea* | 2 | 146 | 39 | 32 | 1 | 5 |  | 57 | 1 | 2 | 9 |
|  | *Pectobacterium* | 1 | 1 | 0 | 0 | 0 | 0 |  | 1 | 0 | 0 | 0 |
|  | *Pseudomonas* | 9 | 345 | 45 | 75 | 48 | 16 |  | 80 | 14 | 43 | 24 |
|  | *Serratia* | 1 | 9 | 0 | 0 | 0 | 9 |  | 0 | 0 | 0 | 0 |
|  | *Sodalis* | 1 | 2 | 0 | 0 | 0 | 0 |  | 1 | 0 | 0 | 1 |
| Bacteroidetes | *Fabibiobacter* | 1 | 22 | 0 | 0 | 5 | 0 |  | 7 | 9 | 1 | 0 |
|  | *Hymenobacter* | 7 | 32 | 3 | 0 | 0 | 12 |  | 0 | 6 | 7 | 4 |
|  | *Mucilaginibacter* | 2 | 1 | 0 | 0 | 0 | 0 |  | 1 | 0 | 0 | 0 |
|  | *Spirosoma* | 1 | 1 | 0 | 0 | 0 | 0 |  | 1 | 0 | 0 | 0 |
| Actinobacteria | *Arthrobacter* | 1 | 1 | 0 | 0 | 1 | 0 |  | 0 | 0 | 0 | 0 |
|  | *Curtobacterium* | 1 | 15 | 3 | 1 | 4 | 2 |  | 0 | 2 | 2 | 1 |
|  | *Friedmanniella* | 1 | 1 | 0 | 0 | 0 | 0 |  | 0 | 0 | 1 | 0 |
|  | *Frigoribacterium* | 1 | 1 | 0 | 0 | 0 | 0 |  | 0 | 0 | 1 | 0 |
|  | *Kineococcus* | 1 | 2 | 0 | 0 | 0 | 0 |  | 0 | 0 | 2 | 0 |
|  | *Labedella* | 1 | 2 | 0 | 0 | 0 | 2 |  | 0 | 0 | 0 | 0 |
|  | *Leifsonia* | 1 | 3 | 0 | 0 | 0 | 0 |  | 0 | 1 | 2 | 0 |
|  | *Microbacterium* | 1 | 2 | 0 | 1 | 0 | 0 |  | 1 | 0 | 0 | 0 |
|  | *Nitriliruptor* | 2 | 2 | 1 | 0 | 0 | 0 |  | 0 | 0 | 0 | 1 |
|  | *Patulibacter* | 1 | 1 | 0 | 0 | 0 | 0 |  | 0 | 0 | 0 | 1 |
|  | *Plantibacter* | 1 | 2 | 1 | 0 | 0 | 0 |  | 0 | 0 | 0 | 1 |
| Firmicutes | *Lactobacillus* | 1 | 25 | 0 | 0 | 0 | 0 |  | 0 | 0 | 0 | 25 |
|  | *Planococcus* | 1 | 1 | 0 | 0 | 0 | 0 |  | 1 | 0 | 0 | 0 |
|  | *Sporacetigenium* | 1 | 1 | 1 | 0 | 0 | 0 |  | 0 | 0 | 0 | 0 |
|  | *Tumebacillus* | 1 | 1 | 0 | 0 | 0 | 0 |  | 1 | 0 | 0 | 0 |
| Tenericutes | *Mesoplasma* | 1 | 3 | 0 | 0 | 0 | 0 |  | 3 | 0 | 0 | 0 |
| Chlorflexi | *Herpetosiphon* | 1 | 1 | 0 | 0 | 0 | 0 |  | 0 | 0 | 0 | 1 |
|  | *Caldilinea* | 1 | 1 | 1 | 0 | 0 | 0 |  | 0 | 0 | 0 | 0 |
| Unclassified |  | 3 | 4 | 1 | 0 | 0 | 0 |  | 1 | 0 | 0 | 2 |
| Total |  | 104 | 1802 | 204 | 205 | 198 | 168 |  | 210 | 263 | 336 | 218 |

^a^Operational taxonomic units (OTUs) were determined at 97% sequence similarity.
