# Supplementary material for: Effect of Streptomycin Treatment on Bacterial Community Structure in the Apple Phyllosphere
Source: PLoS One. 2012 May 21;7(5):e37131. doi: 10.1371/journal.pone.0037131 (PMC3357425; doi:10.1371/journal.pone.0037131)
Supplement: Table S2 — ANOSIM and Mantel values from Bray-Curtis matrix of the bacterial communities at the 100%, 99%, and 97% similarity threshold. The Bonferroni corrected alpha is 0.0056. (DOC) [file pone.0037131.s004.doc]

**Supporting Table 2.** ANOSIM and Mantel values from Bray-Curtis matrix of the bacterial communities at the 100%, 99%, and 97% similarity threshold. The Bonferroni corrected alpha is 0.0056.

A.

B.
