# Supplementary material for: Effect of Streptomycin Treatment on Bacterial Community Structure in the Apple Phyllosphere
Source: PLoS One. 2012 May 21;7(5):e37131. doi: 10.1371/journal.pone.0037131 (PMC3357425; doi:10.1371/journal.pone.0037131)
Supplement: Table S3 — ANOSIM and Mantel values from weighted Unifrac matrix for the bacterial communities excluding Sphingomonas and Pseudomonas species. The Bonferroni corrected alpha is 0.0056. (DOC) [file pone.0037131.s005.doc]

**Supporting Table 3.** ANOSIM and Mantel values from weighted Unifrac matrix for the bacterial communities excluding *Sphingomonas* and *Pseudomonas* species. The Bonferroni corrected alpha is 0.0056.

SUPPLEMENTARY NOTES

The unweighted Unifrac matrix was also calculated for the entire dataset, as well as for the dataset excluding the *Sphingomonas* and *Pseudomonas* species. Neither the PCoA plot, ANOSIM values, nor mantel test values showed a significant effect of any of the variables recorded at the time of sampling.

Furthermore, the taxa shared across all orchard sites were extracted at the phylum, order, class, and genus levels. Unpaired two-tailed Student’s t-tests were done on each taxon to determine any significant effect of long term streptomycin treatment on the bacterial total abundances, the number of OTUs at each site, the relative abundances, the relative number of OTUs, and the number of OTUs normalized to the size of the smallest clone library (i.e., clone library from site LP which had 169 sequences). The lack of significance at any taxonomic level and for any particular taxon confirmed the lack of effect observed using Unifrac and Bray-Curtis.

.
